# Clinicopathological, proliferative, molecular, and prognostic characteristics of differentiated high-grade thyroid carcinoma: a multicenter retrospective study

**DOI:** 10.3389/fonc.2026.1874863

**Published:** 2026-07-08

**Authors:** Wenwen Cui, Lihang Xing, Zhenzhen Li, Xinjun Li, Junzhi Li

**Affiliations:** 1 Department of Pathology, Binzhou People's Hospital Affiliated to Shandong First Medical University, Binzhou, China; 2 Department of Thyroid Surgery, Binzhou People's Hospital Affiliated to Shandong First Medical University, Binzhou, China; 3 Department of Pathology, Shandong Provincial Maternal and Child Health Care Hospital Affiliated to Qingdao University, Jinan, China; 4 Department of Pathology, Tianjin Medical University Cancer Institute and Hospital, National Clinical Research Center for Cancer, Tianjin’s Clinical Research Center for Cancer, Tianjin, China

**Keywords:** BRAF V600E, clinicopathological features, differentiated high-grade thyroid carcinoma, prognosis, thyroid neoplasms

## Abstract

**Objective:**

Differentiated high-grade thyroid carcinoma (DHGTC) is a newly defined pathological subtype introduced in the 5th edition of the World Health Organization (WHO) Classification of Thyroid Tumors in 2022. This study aimed to analyze its clinicopathological characteristics and improve the understanding of this entity among clinicians and pathologists.

**Methods:**

A total of 19 patients with DHGTC from three tertiary medical centers were retrospectively included. Clinical manifestations, histopathological morphology, immunohistochemical findings, and molecular pathological features were analyzed. Postoperative follow-up data were reviewed to evaluate prognosis, and relevant literature was also reviewed.

**Results:**

Among the 19 patients, 9 were male and 10 were female, with a median age of 61 years (range, 24–78 years). The tumors showed marked invasiveness, with 11 cases involving adjacent structures and 13 cases presenting with lymph node metastasis. All cases exhibited high-grade pathological features, characterized by increased mitotic activity and/or tumor necrosis. The Ki-67 proliferation index was ≥10% in all cases, including 10 cases with a Ki-67 index ≥20%, with the highest value reaching 30%. BRAF abnormalities were identified in 9 cases by molecular testing and/or immunohistochemistry, including aberrant immunohistochemical expression and/or molecular confirmation of the BRAF V600E mutation. In addition, two cases harbored a TERT promoter mutation, and one case showed abnormal p53 expression. During follow-up, 4 patients developed recurrence or metastasis, and 2 patients died.

**Conclusions:**

DHGTC is a highly aggressive thyroid malignancy with a poor prognostic tendency. Increased mitotic activity, tumor necrosis, elevated Ki-67 index, and molecular abnormalities such as BRAF alterations are important for diagnosis and prognostic assessment.

## 1 Introduction

Thyroid carcinoma is one of the most common malignant tumors of the endocrine system. Among them, differentiated thyroid carcinoma (DTC), which originates from follicular epithelial cells, accounts for approximately 90%–95% of all cases and mainly includes papillary thyroid carcinoma (PTC) and follicular thyroid carcinoma (FTC) (1–3). Current studies have shown that DTC generally exhibits relatively indolent biological behavior, and most patients achieve favorable long-term survival following standardized surgical treatment and adjuvant therapy (4, 5). However, in recent years, clinical practice and pathological diagnosis have gradually revealed that some thyroid carcinomas with originally differentiated histological features may demonstrate more aggressive behavior and significantly worse clinical outcomes (6). These manifestations include extensive local invasion, early recurrence, and an increased risk of lymph node and distant metastasis, suggesting that traditional histological classification can no longer fully explain their biological heterogeneity (7). Based on this, the World Health Organization (WHO), in the 5th edition of the WHO Classification of Thyroid Tumors published in 2022, officially introduced differentiated high-grade thyroid carcinoma (DHGTC) as a new pathological entity, representing an important update in the stratified diagnosis and risk assessment of follicular cell-derived thyroid neoplasms (8, 9).

DHGTC is a highly aggressive tumor entity that lies between conventional DTC and anaplastic thyroid carcinoma. Its defining feature is the preservation of the histological architecture of DTC while simultaneously exhibiting high-grade pathological features (7, 8). According to the latest WHO classification criteria, these features mainly include tumor necrosis and/or markedly increased mitotic activity, defined as ≥5 mitoses per 2 mm² in hotspot areas (9). In contrast, PDTC is diagnosed according to the Turin criteria, which require a solid, trabecular, or insular growth pattern together with additional diagnostic features, whereas DHGTC retains the architectural features of papillary, follicular, or oncocytic thyroid carcinoma despite exhibiting high-grade features such as tumor necrosis and/or increased mitotic activity. Therefore, the distinction between DHGTC and PDTC primarily relies on architectural patterns rather than proliferative activity alone (7, 10).

Molecular studies suggest that DHGTC is more commonly associated with BRAF V600E-like alterations, whereas PDTC more frequently exhibits RAS-like molecular profiles (7, 8, 11). Additional abnormalities, including TERT promoter mutations and TP53 alterations, may occur in a subset of cases and are associated with more aggressive tumor behavior (12, 13). An elevated Ki-67 proliferation index is frequently observed in DHGTC and may provide supportive information for diagnosis and risk stratification, particularly in morphologically challenging cases (14, 15). Despite increasing recognition of DHGTC, available clinical evidence remains limited because of its rarity. Its clinicopathological characteristics, molecular alterations, treatment response, and long-term prognosis have not yet been fully defined (6–8).

The present study retrospectively analyzed the clinicopathological data of 19 patients with DHGTC from three medical centers, systematically summarizing their histological features, molecular pathological alterations, and follow-up outcomes in combination with the latest literature. This study aimed to further improve the understanding of this newly defined pathological entity and to provide evidence for the standardized diagnosis, risk stratification, and individualized treatment of DHGTC.

## 2 Materials and methods

### 2.1 Study population

This was a multicenter retrospective case series study. A total of 19 patients with pathologically confirmed DHGTC diagnosed between January 1, 2023 and December 31, 2025 at Tianjin Medical University Cancer Institute and Hospital, Binzhou People’s Hospital, and Shandong Provincial Maternal and Child Health Hospital were retrospectively enrolled, and all cases were reviewed according to the 2022 WHO Classification of Thyroid Tumours.

All cases were independently re-reviewed and confirmed by at least two senior pathologists according to the diagnostic criteria of the 5th edition of the World Health Organization (WHO) Classification of Thyroid Tumors (2022) (9). The diagnostic criteria were defined as the presence of tumor necrosis and/or ≥5 mitoses per 2 mm² in hotspot areas on the basis of the histomorphology of differentiated thyroid carcinoma.

This study was approved by the Institutional Ethics Committee (Approval No.: YXKYLL_20260406) of Binzhou People’s Hospital, Binzhou, China. The requirement for informed consent was waived due to the retrospective nature of the study. Patient data were anonymized, and no identifiable personal information is included. All procedures were conducted in accordance with the ethical standards of the institutional research committee and the Declaration of Helsinki.

### 2.2 Clinical data collection

Clinical and pathological data were collected for all patients, including demographic information (age and sex), tumor-related characteristics (tumor location, maximum tumor diameter, and extent of invasion), metastatic status (central and lateral cervical lymph node metastasis), pathological parameters (mitotic count, tumor necrosis, and Ki-67 proliferation index), and molecular pathological findings (including BRAF, TERT, and p53 gene testing and/or immunohistochemical results). Treatment and follow-up data were also collected, mainly including surgical procedures, postoperative radioactive iodine (^131I) therapy, local ablation, targeted therapy, and clinical outcomes.

### 2.3 Pathological evaluation

All cases underwent routine paraffin embedding, sectioning, and hematoxylin–eosin (H&E) staining for histopathological examination. The histological subtype, local invasion, tumor necrosis, and mitotic activity were systematically evaluated. Mitotic figures were counted in the most proliferative hotspot area, with the region containing the highest density of mitoses selected. The total number of mitotic figures within a continuous area of 2 mm² was recorded as the mitotic count per 2 mm². All slides were independently reviewed by two senior pathologists, and mitotic counts were performed separately. In cases of discrepancy, a consensus was reached through joint review and discussion.

For selected cases in which mitotic figures were difficult to identify because of interference from apoptotic bodies or nuclear debris, PHH3 immunohistochemical staining was additionally performed to assist mitotic figure recognition and counting.

The Ki-67 proliferation index was assessed by immunohistochemistry, and the percentage of positively stained tumor cells was evaluated in hotspot areas. Molecular testing was not uniformly available for all patients. BRAF status was assessed by immunohistochemistry and/or molecular testing according to institutional practice. TERT promoter mutation analysis and p53 immunohistochemical evaluation were performed in selected cases when available.

### 2.4 Follow-up

Follow-up data regarding postoperative recurrence, metastasis, and survival outcomes were collected through outpatient records, inpatient medical record systems, and telephone follow-up. The follow-up period was defined as the interval from the date of initial diagnosis or surgical treatment to the date of last follow-up or death. Survival outcomes included overall survival (OS) and progression-free survival (PFS). Death was defined as the event for OS, whereas local recurrence, distant metastasis, disease progression, or death was defined as the event for PFS. For patients lost to follow-up, the date of the last valid follow-up was recorded.

### 2.5 Statistical analysis

Statistical analyses were performed using SPSS version 29.0 (IBM Corp., Armonk, NY, USA), and Kaplan–Meier survival curves and related figures were generated using GraphPad Prism version 10.0. Continuous variables were expressed as mean ± standard deviation or median (range), whereas categorical variables were presented as numbers and percentages. OS and PFS were analyzed using the Kaplan–Meier method, and survival curves were plotted accordingly. Given the small sample size and retrospective case-series design of the present study, the analysis was mainly based on descriptive statistics.

## 3 Results

The overall demographic, clinicopathological, molecular, treatment, and outcome characteristics of the cohort are summarized in **Table 1**.

**Table 1:** Summary of demographic, clinicopathological, molecular, treatment, and outcome characteristics of the cohort.

Variable	Value
Number of patients	19
Age, median (range)	61 (24–78) years
Sex (Male)	9 (47.4%)
Tumor size ≥2 cm	11 (57.9%)
Local invasion	11 (57.9%)
Lymph node metastasis	13 (68.4%)
Tumor necrosis	3 (15.8%)
Ki-67	
≥10%	18/18 available cases (100%)
≥20%	10/18 available cases (55.6%)
BRAF results available	11 (57.9%)
Abnormalities	9/11 (81.8%)
wild-type	2/11 (18.2%)
TERT promoter mutation	2 patients
Abnormal p53 expression	1 patient
Surgery	17 (89.5%)
Radioactive iodine therapy	13 (68.4%)
Recurrence or metastasis	4 (21.1%)
Death due to disease	2 (10.5%)
Median follow-up	12 (3–29) months

### 3.1 General clinical characteristics

A total of 19 patients with differentiated high-grade thyroid carcinoma (DHGTC) were included in this study, comprising 9 males and 10 females. The median age was 61 years (range, 24–78 years), with a mean age of 59.0 ± 15.3 years. Eighteen patients (94.7%) were older than 30 years, indicating that DHGTC mainly occurred in middle-aged and elderly patients. The primary lesions were mainly located in unilateral or bilateral thyroid lobes, with additional cases involving postoperative residual lobe recurrence lesions and metastatic lesions. The maximum tumor diameter ranged from 0.4 to 5.3 cm, among which 11 cases had tumors ≥2 cm, and only one case was a micro-lesion (0.4 cm).

**Figure 1:**
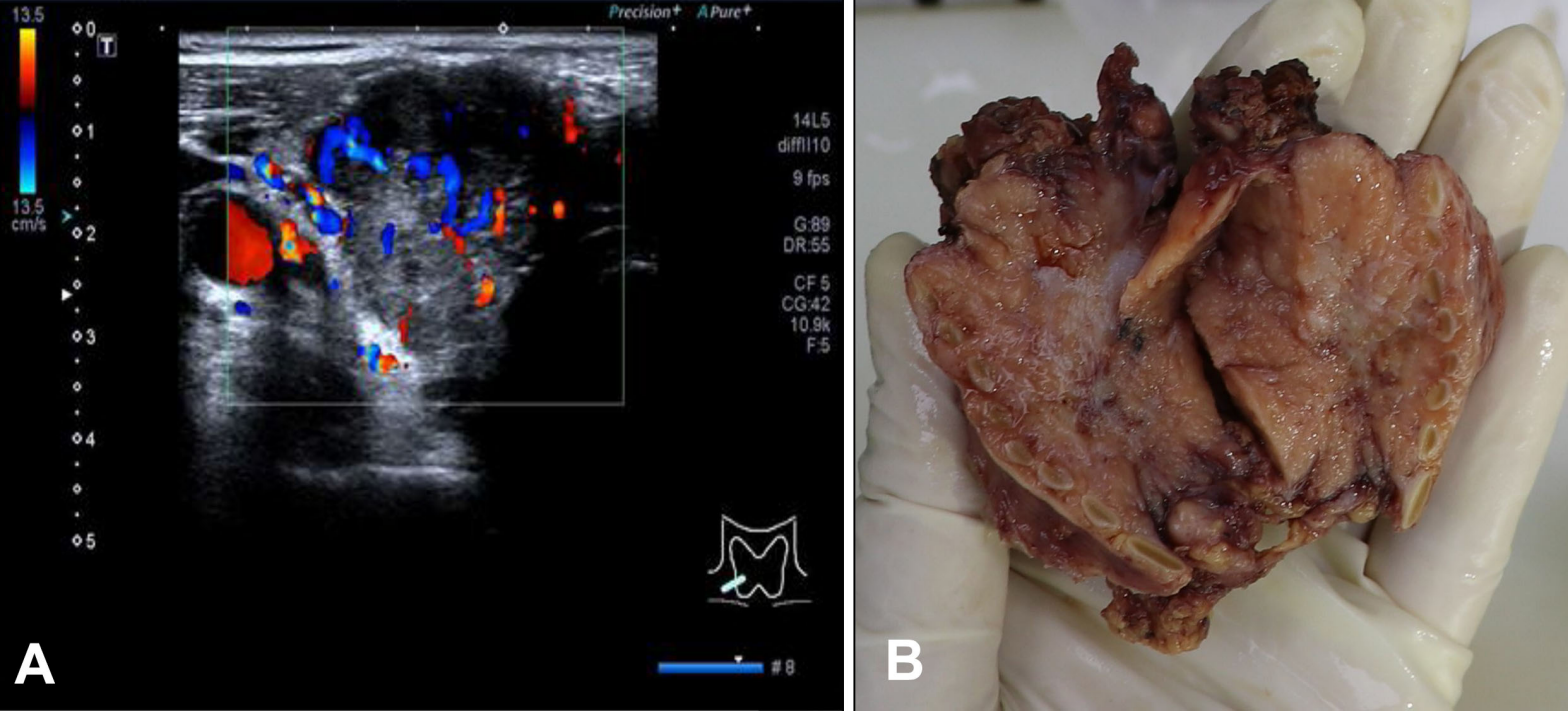
Representative imaging and gross pathological findings of differentiated high-grade thyroid carcinoma (DHGTC). **(A)** Preoperative color Doppler ultrasonography showing a hypoechoic thyroid lesion with abundant intralesional and peripheral blood flow signals. **(B)** Gross specimen of the thyroid gland and tracheal tissue after surgery, showing a gray-red infiltrative mass invading the full thickness of the tracheal wall and involving the cartilage ring.

To visually demonstrate the clinical and gross pathological features of DHGTC, one representative case was selected (**Figure 1**). Preoperative thyroid color Doppler ultrasonography revealed a hypoechoic nodule with abundant internal and peripheral blood flow signals, suggesting a rich blood supply to the lesion (**Figure 1A**). Gross examination of the postoperative thyroid and tracheal specimen showed a gray-red mass measuring approximately 4.5 × 3.5 × 3.0 cm located in the inner mucosa and subcartilaginous region of the tracheal cartilage ring. The lesion had ill-defined margins and a firm texture, showing obvious infiltrative growth involving the full thickness of the tracheal wall and cartilage ring, indicating strong local invasiveness (**Figure 1B**).

### 3.2 Local invasion and lymph node metastasis

Local invasion was common, with 11 cases (57.9%) showing obvious infiltrative growth involving adjacent structures, including extrathyroidal soft tissue, trachea, skeletal muscle, nerves, vessels, and parotid gland. Specifically, 2 cases involved the trachea, 2 involved skeletal or striated muscle, 1 involved nerves, 1 involved the parotid gland, and 6 involved cervical soft tissue and extrathyroidal adipose tissue.

Lymph node metastasis was identified in 13 cases (68.4%), involving both central and lateral cervical lymph node compartments. Cases with a high metastatic burden included one patient with 15/41 positive left cervical lymph nodes and one patient with 11/22 positive central and bilateral cervical lymph nodes, indicating a strong tendency for lymphatic spread.

### 3.3 Pathological and molecular pathological features

On hematoxylin–eosin (H&E) staining, all 19 DHGTC cases exhibited definite high-grade histological features and fulfilled the WHO 2022 diagnostic criteria.

**Figure 2:**
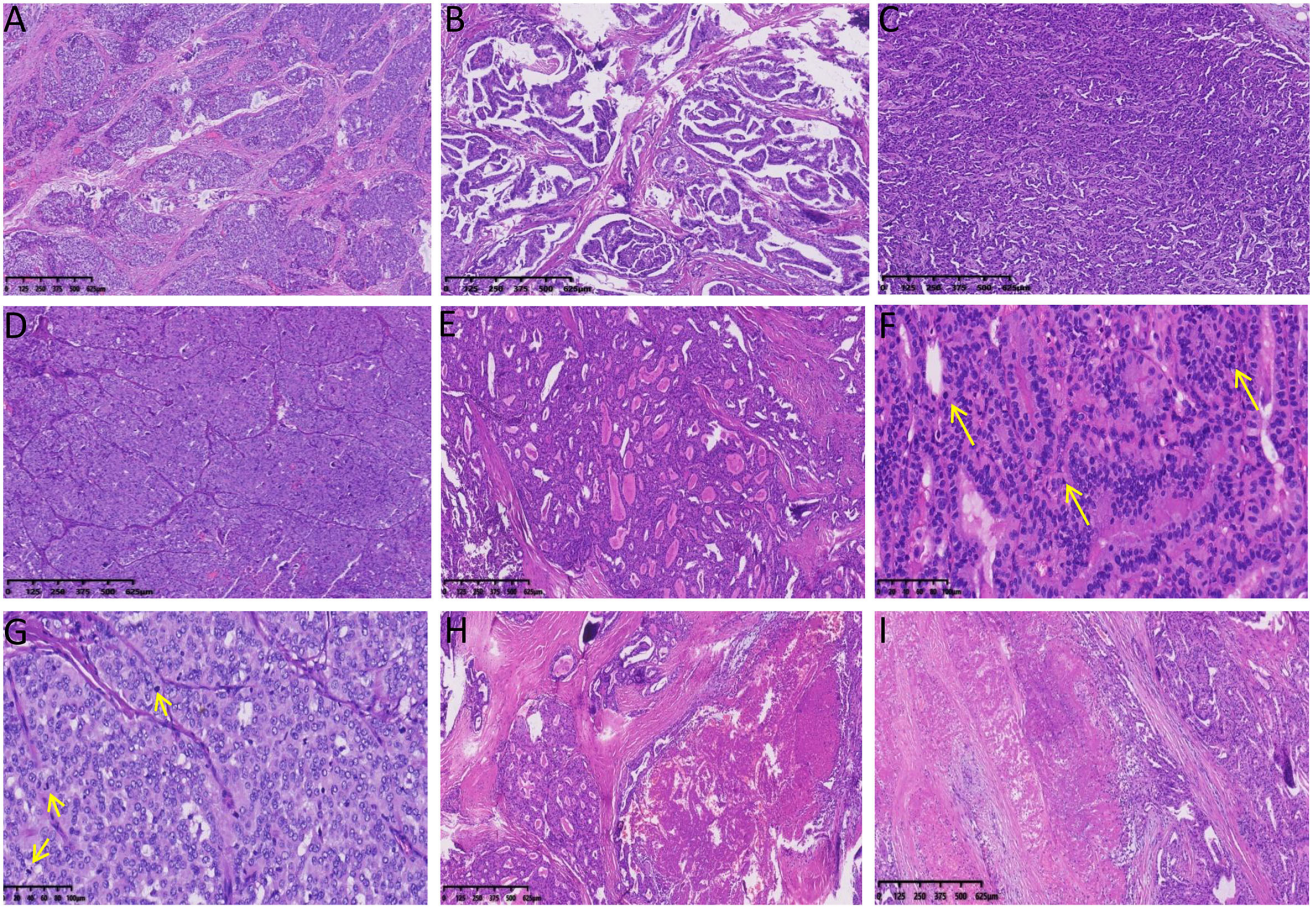
Hematoxylin and eosin (H&E) morphological features of differentiated high-grade thyroid carcinoma (DHGTC). **(A)** Tumor cells arranged in nested clusters (×40). **(B)** Papillary growth pattern of tumor cells (×40). **(C)** Trabecular and slit-like arrangement of tumor cells (×40). **(D)** Sheet-like and solid growth pattern (×40). **(E)** Follicle-like structure formation (×40). **(F)** Glandular-like arrangement of tumor cells (×40). **(G, H)** Numerous mitotic figures are observed (arrows, ×200). **(I)** Tumor necrosis with adjacent viable tumor cells (×40).

The tumor cells showed marked morphological heterogeneity and presented with multiple architectural patterns, including nested (**Figure 2A**), papillary (**Figure 2B**), trabecular and slit-like (**Figure 2C**), sheet-like or solid (**Figure 2D**), follicular-like (**Figure 2E**), and glandular-like structures (**Figure 2F**). These findings indicate that the tumors retained certain structural features of differentiated thyroid carcinoma while simultaneously exhibiting obvious high-grade histological changes.

All cases showed increased mitotic activity, and the mitotic counts in all cases reached the WHO diagnostic threshold (≥5 mitoses per 2 mm²), with 4 cases showing markedly elevated mitotic activity. Numerous mitotic figures were observed in **Figures 2F, G** (indicated by yellow arrows), suggesting active tumor proliferation.

In addition, 3 cases (15.8%) showed definite tumor necrosis (**Figures 2H, I**), manifested as focal or patchy eosinophilic structureless necrotic areas surrounded by viable tumor cells.

The Ki-67 proliferation index ranged from 10% to 30%, with all cases showing values ≥10%. Among them, 10 cases (52.6%) had Ki-67 values ≥20%, and 2 cases (10.5%) reached 30%, further supporting the high proliferative activity of DHGTC. Overall, DHGTC demonstrated both the structural features of differentiated thyroid carcinoma and the morphological characteristics of high-grade malignancy under microscopy, with active mitotic activity and tumor necrosis serving as the main diagnostic criteria.

**Figure 3:**
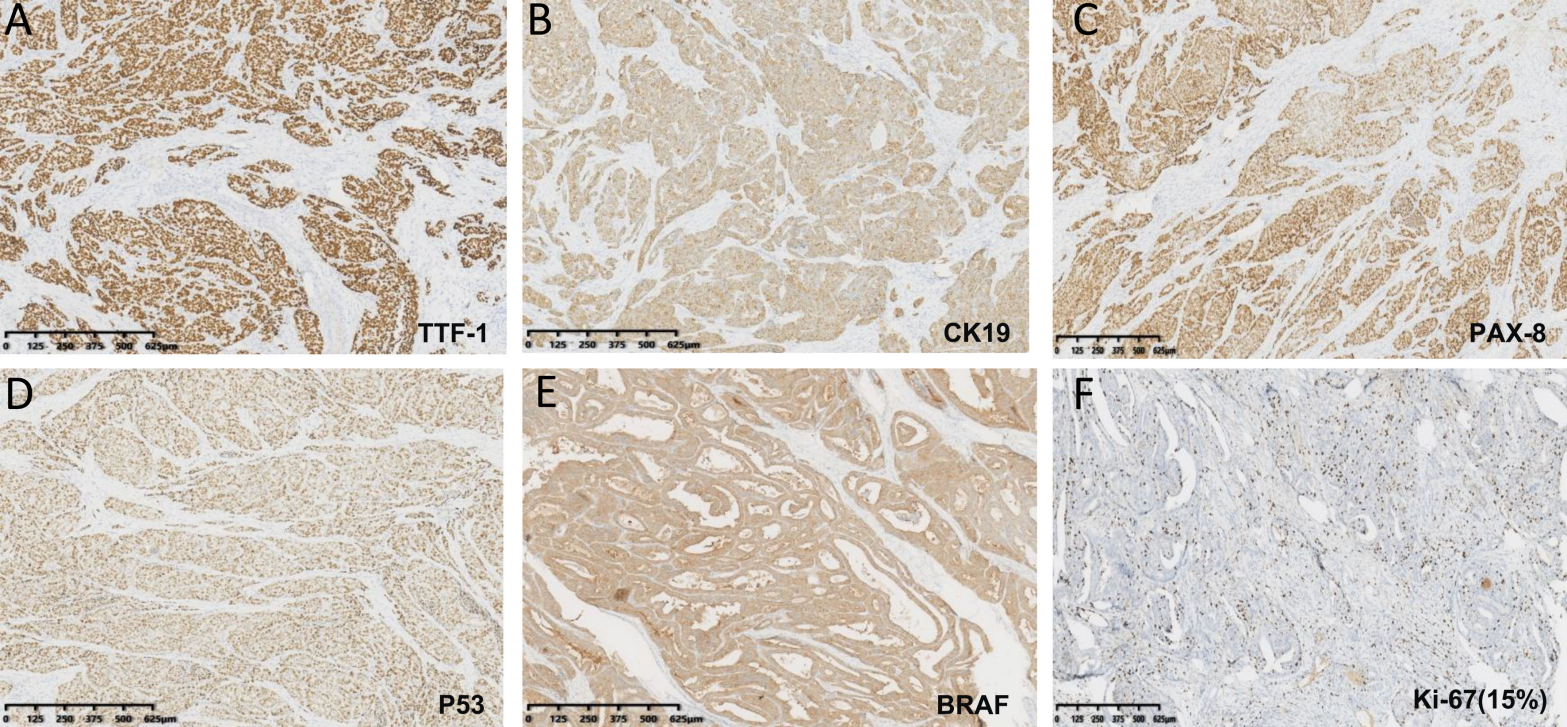
Immunohistochemical features of differentiated high-grade thyroid carcinoma (DHGTC). **(A)** TTF-1. **(B)** CK19. **(C)** PAX8. **(D)** Positive p53 expression. **(E)** Positive BRAF staining. **(F)** Ki-67 proliferation index of approximately 15%. All images were obtained at ×200 magnification.

Immunohistochemical staining of representative cases showed diffuse expression of thyroid lineage markers, including TTF-1, CK19, and PAX8 (**Figures 3A–C**), supporting their follicular epithelial origin. Partial tumor cells showed positive p53 expression (**Figure 3D**), suggesting underlying molecular abnormalities. BRAF immunohistochemical staining was positive (**Figure 3E**), and together with molecular findings supported the presence of driver gene alterations. The Ki-67 proliferation index was approximately 15% (**Figure 3F**), indicating high proliferative activity. Taken together with the histological morphology, these immunophenotypic findings were consistent with the pathological features of DHGTC.

**Figure 4:**
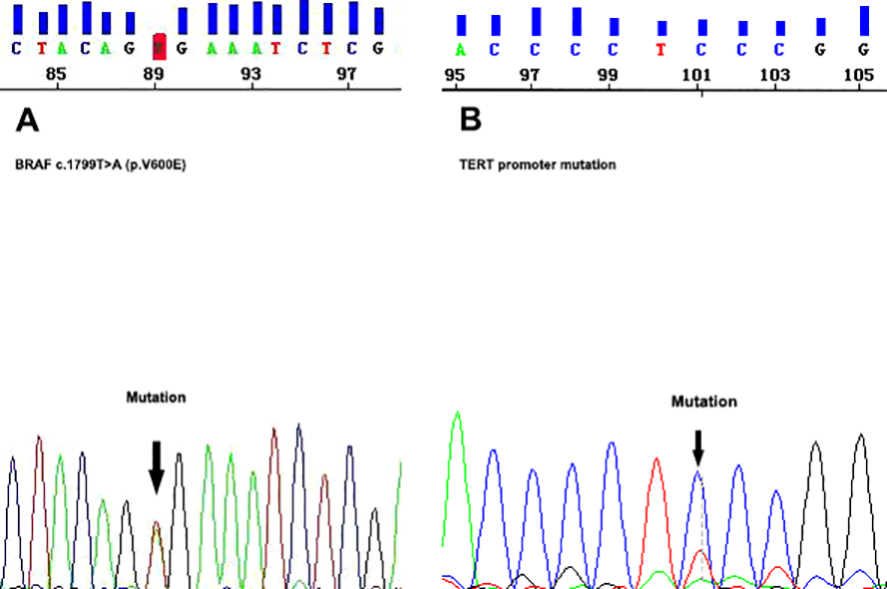
Molecular pathological findings of differentiated high-grade thyroid carcinoma (DHGTC). **(A)** Sanger sequencing demonstrating a heterozygous BRAF V600E (c.1799T>A) mutation with a characteristic double-peak signal at the mutation site. **(B)** Detection of a TERT promoter mutation in two representative case.

Molecular data were available for a subset of patients. Among the 11 patients with available BRAF results, 9 (81.8%) showed BRAF abnormalities, including positive or weakly positive immunohistochemical staining and/or molecular confirmation of the BRAF V600E mutation. Two patients harbored TERT promoter mutations, and one patient showed aberrant p53 expression. In some cases, Sanger sequencing further confirmed a heterozygous base substitution in the hotspot region of BRAF exon 15, manifested as a double-peak signal at the mutation site, consistent with the BRAF V600E (c.1799T>A) mutation (**Figure 4A**).

In addition, two cases (10.5%) harbored a TERT promoter mutation (**Figure 4B**), and one case (5.3%) showed abnormal p53 expression. Meanwhile, some cases remained BRAF-negative, suggesting a certain degree of molecular heterogeneity in DHGTC and indicating that, in addition to the classical BRAF-driven event, other molecular pathways may also be involved.

### 3.4 Treatment and follow-up outcomes

A total of 17 patients underwent surgical treatment (**Table 2**). The extent of surgery varied according to tumor location, local invasion, and disease status, and included total thyroidectomy, hemithyroidectomy, completion thyroidectomy, cervical lymph node dissection, partial tracheal resection, and palliative surgery. Two patients did not undergo definitive surgical resection and were diagnosed by biopsy. In addition, 13 patients received postoperative radioactive iodine (^131I) therapy, whereas selected recurrent cases underwent local ablation, repeat lymph node dissection, or targeted therapy. One patient (Case 2) with recurrent BRAF-mutant DHGTC received dabrafenib after postoperative recurrence; however, the therapeutic response was limited. Detailed surgical procedures and TNM staging information for individual patients are provided in **Table 2**.

**Table 2:** Individual clinicopathological, molecular, treatment, and outcome characteristics of 19 patients with DHGTC.

Case	Age	Sex	Tumor site/size	Local invasion	LN metastasis	Ki-67 (%)	High-grade features	Molecular findings	Surgery	Adjuvant therapy	Outcome	TNM
1	40	M	Left remnant lobe, 4.0 cm	Trachea	No	20	Mitoses >5/2 mm²	BRAF WT	CT + LND	RAI	NED, 28 mo	pT4aN1bM0
2	61	M	Right thyroid, 4.5 cm	Trachea, muscle	1/1	20	Mitoses >5/2 mm²	BRAF+, TERT+	HT + LND + PTR	RAI + DAB	Recurrence, 14–15.5 mo	rT4aN1bM0
3	57	F	Right cervical LN, 1.7 cm	No	8/12	10–15	Frequent mitoses	BRAF IHC+	TT + LND + CND	RAI	NED, 18 mo	rT2N1bM0
4	59	M	Left cervical soft tissue, 2.5 cm	No	No	15–20	Mitoses	BRAF IHC weak+	LND	RAI	LTFU, 12 mo	pTXN1bM0
5	72	F	Left parotid tail, 2.0 cm	Parotid	1/3	15–20	Mitoses >5/2 mm²	BRAF+	CT + BND	RAI	LTFU, 6 mo	pT4N1bM0
6	73	M	Right thyroid, 1–2 cm	Perithyroid tissue	11/22	10	Necrosis	BRAF IHC+	TT + CND + BND	RAI	NED, 18 mo	pT4aN1bM0
7	68	F	Right thyroid, 5.2 cm	Extrathyroid fat	Yes	10	Necrosis	NA	HT	RAI + Ablation	Stable, 18 mo	pT4aNxM0
8	75	F	Neck mass, 4.7 cm	Suspected trachea	NA	10	Necrosis	BRAF IHC weak+	CNB only	None	DOD, 3 mo	pT4aNxM0
9	67	M	Right + isthmus, 2.0 cm	Nerve	2/4	20	Mitoses >5/2 mm²	NA	TT + CND	RAI	NED, 29 mo	pT2N1aM0
10	61	F	Right thyroid, 5.3 cm	Capsule, muscle	NA	30	>20/10 HPF	NA	Palliative thyroidectomy	RAI	Suspected recurrence, 24 mo	pT3N0M0
11	71	F	Left thyroid, 3.0 cm	No	0/1	10	16/10 HPF	NA	HT + CND	RAI	NED, 22 mo	pT2N0M0
12	24	F	Left thyroid, 0.4 cm	Capsule	No	15	8/2 mm²	p53+, BRAF NA	TT + CND	RAI	NED, 10 mo	pT1aN0M0
13	42	F	Left thyroid, 2.1 cm	No	1/4	10	6/2 mm²	BRAF WT	TT + CND	RAI	NED, 10 mo	pT2N1aM0
14	38	F	Right thyroid	Capsule, vessel	No	NA	>5/HPF	NA	HT	None	NED, 6 mo	pT2NxM0
15	61	M	Left neck soft tissue	Vein wall + LN	Yes	25	>10/HPF	NA	Soft tissue resection + vascular repair	None	Lung metastasis, 12 mo	pT4aN1bM0
16	66	M	Cervical LN	Cervical LN	Yes	20	>5/HPF	NA	CNB only	None	DOD, 12 mo	pTXN1M0
17	78	M	Recurrent left cervical LN	LN	4/7	30	>5/HPF	BRAF IHC+	LND	None	Stable, 6 mo	rT4aN1bM0
18	70	F	Recurrent left cervical LN	LN + soft tissue	4/9	20	>5/HPF	BRAF+, TERT+	LND	None	NED, 8 mo	rTXN1bM0
19	38	M	Left thyroid recurrence	Left cervical LN	15/41	15	>5/HPF	BRAF+, IHC weak+	HT + LND	None	NA	pT2N1bM0

F, Female; M, Male; y, year; LN, Lymph Node; HPF, High-Power Field; NED, No evidence of disease; DOD, Died of disease; LTFU, Lost to follow-up; TT, Total thyroidectomy; HT, Hemithyroidectomy; CT, Completion thyroidectomy; CND, Central neck dissection; DAB, Dabrafenib; LND, Lateral neck dissection; BND, Bilateral neck dissection; PTR, Partial tracheal resection; RAI, Radioactive iodine therapy; CNB, Core needle biopsy. NA, Not available.

The median follow-up time was 12 months (range, 3–29 months). At the last follow-up, 9 patients (47.4%) showed no definite recurrence or metastasis. Postoperative local recurrence or distant metastasis occurred in 4 cases (21.1%), including one laryngeal metastasis, one pulmonary metastasis, and two local recurrences. Two patients (10.5%) died, one within 3 months after biopsy and the other within 1 year. Four patients (21.1%) were lost to follow-up.

**Figure 5:**
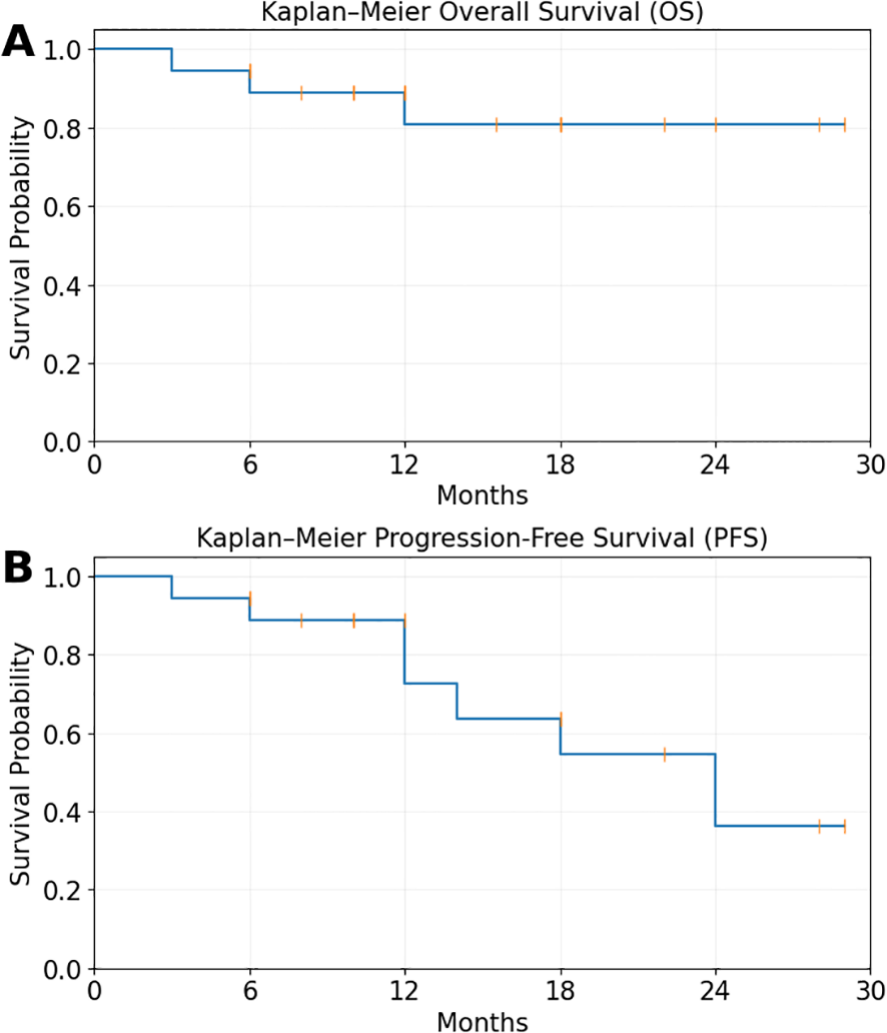
Kaplan–Meier survival curves of patients with differentiated high-grade thyroid carcinoma (DHGTC). **(A)** Overall survival (OS). **(B)** Progression-free survival (PFS). Because only a limited number of patients remained at risk beyond 24 months, survival estimates after this time point should be interpreted with caution.

Kaplan–Meier survival analysis showed (**Figure 5**) that the overall survival (OS) rate remained at a relatively high level during the early follow-up period, being approximately 81% at 12 months, and remained around 80% at the last follow-up (approximately 29 months) (**Figure 5A**). In contrast, the progression-free survival (PFS) curve showed a continuous downward trend, being approximately 73% at 12 months, decreasing to 55% at 18 months, and further declining to approximately 36% after 24 months (**Figure 5B**).

## 4 Discussion

DHGTC is an important pathological entity newly introduced in the 5th edition of the WHO Classification of Thyroid Tumors in 2022, representing a highly aggressive follicular cell-derived malignancy that lies between conventional differentiated thyroid carcinoma (DTC) and anaplastic thyroid carcinoma (8). Compared with conventional papillary thyroid carcinoma (PTC), DHGTC retains the histological architecture of differentiated thyroid carcinoma while exhibiting higher proliferative activity, stronger local invasiveness, and poorer clinical outcomes (6, 16). Therefore, its accurate identification is of great significance for clinical decision-making and prognostic assessment.

A total of 19 patients with DHGTC were included in this study. The mean age was 59.0 ± 15.3 years, and most patients were middle-aged or elderly, which is generally consistent with previous reports indicating that DHGTC is more common in patients over 50 years of age (6, 7, 16). Notably, one patient in this cohort was only 24 years old, with a lesion measuring only 0.4 cm, suggesting that DHGTC is not restricted to elderly patients and that large tumor volume is not a prerequisite for diagnosis (see **Supplementary Figure 1**). This finding has important clinical implications, indicating that even in young patients or micro-lesions, the possibility of DHGTC should be considered when pathology shows active mitotic activity or an elevated proliferation index.

From the histological perspective, all cases in this study met the WHO diagnostic criteria, namely, tumor necrosis and/or ≥5 mitoses per 2 mm² in hotspot areas on the basis of differentiated thyroid carcinoma morphology (8, 17). All cases showed increased mitotic activity, and 3 cases demonstrated definite tumor necrosis, further confirming the central role of mitotic activity and necrosis in the diagnosis of DHGTC. It is noteworthy that although some cases showed high-grade architectural patterns such as solid and trabecular growth, all retained definite nuclear or structural features of differentiated thyroid carcinoma and therefore did not fall within the morphological spectrum of pure poorly differentiated thyroid carcinoma (PDTC), ultimately supporting the diagnosis of DHGTC. Unlike PDTC, DHGTC still retains the basic architecture of papillary or follicular carcinoma, which is one of the most easily overlooked points in routine clinical pathology practice. Traditional thyroid pathology diagnosis often places greater emphasis on the nuclear features of PTC, capsular invasion, and vascular tumor thrombi, whereas systematic mitotic counting is often insufficiently emphasized (18–20). Our findings suggest that routine hotspot mitotic counting should be performed in all follicular cell-derived thyroid malignancies, particularly in cases with the tall-cell variant, recurrent lesions, or marked architectural atypia, to avoid missed diagnosis of DHGTC.

The Ki-67 proliferation index was ≥10% in all cases, ranging from 10% to 30%, with 10 cases ≥20%, indicating significant proliferative activity in DHGTC. Although Ki-67 has not yet been included as an independent diagnostic criterion by the WHO, increasing evidence suggests that it has important value in auxiliary diagnosis and prognostic stratification (21–23). Our findings are consistent with previous studies indicating that elevated Ki-67 is usually associated with higher invasiveness and poorer prognosis. Particularly in morphologically borderline cases, the combined use of Ki-67 and PHH3 staining may help improve the accuracy of mitotic figure identification.

Regarding invasive behavior, 11 cases (57.9%) in this study showed obvious local invasion, and 13 cases (68.4%) had lymph node metastasis, indicating a strong tendency for local invasion and lymphatic spread. Some cases exhibited invasion of the trachea, skeletal muscle, nerves, and soft tissues, and even recurrence in the parotid region, demonstrating biological behavior significantly more aggressive than that of classical PTC. In particular, several cases represented recurrent lesions occurring many years after surgery, including recurrence at 6, 10, and even 16 years postoperatively, suggesting that DHGTC may represent a stage of malignant progression during the long-term evolution of a subset of DTCs. This observation is consistent with the previously proposed theory of “progression of high-risk differentiated carcinoma toward a high-grade phenotype” (6, 7, 11).

The molecular pathological findings further support the biological heterogeneity of DHGTC. In the present study, BRAF abnormalities were identified in 9 of 11 patients with available BRAF results, including positive or weakly positive immunohistochemical staining and/or molecular confirmation of the BRAF V600E mutation. Two patients harbored TERT promoter mutations, and one patient showed aberrant p53 expression. Previous studies have suggested that BRAF V600E is one of the most common driver events in DHGTC, whereas TERT promoter mutations and TP53 alterations are associated with tumor progression and adverse clinical outcomes. However, molecular testing was not uniformly performed across all patients, and the number of cases with TERT promoter mutations and abnormal p53 expression was limited. Therefore, the prevalence and clinical significance of these alterations could not be reliably assessed in the present cohort. In addition, two patients were identified as BRAF wild-type, supporting the molecular heterogeneity of DHGTC and suggesting that a diagnosis of DHGTC should not be excluded solely on the basis of negative BRAF status.

From the prognostic perspective, 4 patients (21.1%) developed local recurrence or distant metastasis during follow-up, and 2 patients (10.5%) died, indicating an overall prognosis clearly worse than that of conventional PTC. The distribution of follow-up outcomes further demonstrates that although approximately half of the patients had no recurrence or metastasis during the follow-up period, the proportions of recurrence, metastasis, and death remained non-negligible, indicating a relatively high risk of disease progression. These findings are generally consistent with previous studies on high-grade non-anaplastic thyroid carcinoma (HGTC) (19). Relevant reviews have suggested that HGTC, including PDTC and DHGTC, exhibits biological behavior intermediate between DTC and anaplastic thyroid carcinoma, with moderate to high aggressiveness, and that approximately 20%–50% of patients may develop local progression or distant metastasis, with long-term survival rates significantly lower than those of conventional DTC (24). In addition, some patients respond poorly to radioactive iodine therapy, further suggesting a tendency toward dedifferentiation and treatment resistance. Compared with previous reports, the overall follow-up outcomes in this study were relatively favorable, which may be related to the limited sample size, relatively short follow-up duration, and the fact that some cases were at a relatively early stage. Nevertheless, adverse prognostic factors such as local invasion, lymph node metastasis, and elevated Ki-67 were still observed. Notably, several patients experienced rapid disease progression and even death within a short period, highlighting the marked clinical heterogeneity of DHGTC. Taken together, our findings further support the concept proposed in the Introduction that the prognosis of DHGTC lies between DTC and anaplastic carcinoma, and that it should be regarded as a high-risk thyroid tumor requiring enhanced risk stratification, more aggressive multimodal treatment strategies, and closer follow-up.

In terms of treatment, there is currently no unified consensus for DHGTC. Most patients in this cohort underwent complete surgical resection combined with radioactive iodine (^131I) therapy, while some recurrent cases received local ablation, repeat lymph node dissection, and targeted therapy. Based on the current findings, complete surgical resection remains the most critical treatment modality (4, 25). For cases with TERT or p53 abnormalities, significantly elevated Ki-67, or recurrent/metastatic disease, whether more aggressive postoperative adjuvant treatment should be adopted warrants further investigation (13, 22, 26).

This study has several limitations. First, the sample size was relatively small. Although this represents one of the larger multicenter series of DHGTC reported to date, the rarity of this entity limited the number of available cases. Consequently, subgroup analyses and multivariable prognostic modeling were not feasible, and the statistical power of the study remains limited. Second, some patients were lost to follow-up, resulting in insufficient long-term survival analysis. In addition, molecular testing was not uniformly performed across all cases. BRAF status was assessed using either immunohistochemistry or molecular testing according to institutional practice, whereas TERT promoter mutation and p53 analyses were available only in a subset of patients. Therefore, the reported frequencies of molecular alterations should be interpreted with caution and may not accurately reflect their true prevalence in DHGTC. Furthermore, the median follow-up time in this study was 12 months, which is relatively short and may underestimate delayed recurrence or distant metastasis events. Therefore, the findings should be interpreted with caution. Larger multicenter prospective cohorts and registry-based studies incorporating appropriate control groups, such as conventional PTC or PDTC, and longer follow-up periods are needed to further validate our observations and clarify the molecular characteristics and optimal management strategies of DHGTC.

Overall, this study further confirms that DHGTC is a novel thyroid malignant tumor entity characterized by high aggressiveness and a poor prognostic tendency. In pathological diagnosis, comprehensive evaluation of mitotic activity, tumor necrosis, and the Ki-67 proliferation index should be highly emphasized to improve its recognition rate and optimize clinical management.

## Data Availability

The original contributions presented in the study are included in the article/**Supplementary Material**. Further inquiries can be directed to the corresponding authors.

## Supplementary material

Supplementary Figure 1 Histopathological features of the 24-year-old patient with differentiated high-grade thyroid carcinoma (DHGTC). **(A)** H&E staining showing frequent mitotic figures at ×200 magnification, with a low-power inset view (×20) demonstrating that the tumor was confined within the thyroid lobe. **(B)** Diffuse positive CK19 expression by immunohistochemistry. **(C)** PHH3 staining demonstrating numerous mitotic figures in a high-power field (×400). **(D)** Ki-67 staining showing a relatively high proliferative index.

## References

[1] van Houten P Netea-Maier RT Smit JW . Differentiated thyroid carcinoma: an update. Best Pract Res Clin Endocrinol Metab. (2023) 37:101687. doi: 10.1016/j.beem.2022.101687 36002346

[2] McLeod DS . Current concepts and future directions in differentiated thyroid cancer. Clin Biochemist Rev. (2010) 31:9. PMC2826267 20179793

[3] Tumino D Frasca F Newbold K . Updates on the management of advanced, metastatic, and radioiodine refractory differentiated thyroid cancer. Front Endocrinol. (2017) 8:312. doi: 10.3389/fendo.2017.00312 29209273 PMC5702018

[4] Ringel MD Sosa JA Baloch Z Bischoff L Bloom G Brent GA . 2025 American Thyroid Association management guidelines for adult patients with differentiated thyroid cancer. Thyroid®. (2025) 35:841–985. doi: 10.1177/10507256251363120 40844370 PMC13090833

[5] Luis P-O Lucía M-J Hugo R-C Ramiro R-M Stalin C-Q . Differentiated thyroid carcinoma long‐term prognostic factors. Int J Surg Oncol. (2024) 2024:1067447. doi: 10.1155/2024/1067447 39291250 PMC11407879

[6] Resta IT Gubbiotti M Montone K Livolsi V Baloch Z . Differentiated high grade thyroid carcinomas: diagnostic consideration and clinical features. Hum Pathol. (2024) 144:53–60. doi: 10.1016/j.humpath.2024.01.002 38244615

[7] Harahap AS Roren RS Imtiyaz S . A comprehensive review and insights into the new entity of differentiated high-grade thyroid carcinoma. Curr Oncol. (2024) 31:3311–28. doi: 10.3390/curroncol31060252 38920735 PMC11203239

[8] Baloch ZW Asa SL Barletta JA Ghossein RA Juhlin CC Jung CK . Overview of the 2022 WHO classification of thyroid neoplasms. Endocr Pathol. (2022) 33:27–63. doi: 10.1007/s12022-022-09707-3 35288841

[9] Jung CK Bychkov A Kakudo K . Update from the 2022 World Health Organization classification of thyroid tumors: a standardized diagnostic approach. Endocrinol Metab. (2022) 37:703–18. doi: 10.3803/enm.2022.1553 36193717 PMC9633223

[10] Volante M Collini P Nikiforov YE Sakamoto A Kakudo K Katoh R . Poorly differentiated thyroid carcinoma: the Turin proposal for the use of uniform diagnostic criteria and an algorithmic diagnostic approach. Am J Surg Pathol. (2007) 31:1256–64. doi: 10.1097/pas.0b013e3180309e6a 17667551

[11] Landa I Ibrahimpasic T Boucai L Sinha R Knauf JA Shah RH . Genomic and transcriptomic hallmarks of poorly differentiated and anaplastic thyroid cancers. J Clin Invest. (2016) 126:1052–66. doi: 10.1172/jci85271 26878173 PMC4767360

[12] Riesco-Eizaguirre G . BRAF V600E in thyroid cancer: navigating prognostic uncertainty and therapeutic opportunity. Eur Thyroid J. (2025) 14:e250225. doi: 10.1530/etj-25-0225 41368991 PMC12741945

[13] Tan G Jin B Qian X Wang Y Zhang G Agyekum EA . TERT promoter mutations contribute to adverse clinical outcomes and poor prognosis in radioiodine refractory differentiated thyroid cancer. Sci Rep. (2024) 14:23719. doi: 10.1038/s41598-024-75087-9 39390090 PMC11467215

[14] Hellgren LS Stenman A Paulsson JO Höög A Larsson C Zedenius J . Prognostic utility of the Ki-67 labeling index in follicular thyroid tumors: a 20-year experience from a tertiary thyroid center. Endocr Pathol. (2022) 33:231–42. doi: 10.1007/s12022-022-09714-4 35305239 PMC9135869

[15] Kakudo K Wakasa T Ohta Y Yane K Ito Y Yamashita H . Prognostic classification of thyroid follicular cell tumors using Ki-67 labeling index: risk stratification of thyroid follicular cell carcinomas. Endocrine J. (2015) 62:1–12. doi: 10.1507/endocrj.ej14-0293 25195708

[16] Jeong SI Kim W Yu HW Choi JY Ahn CH Moon JH . Incidence and clinicopathological features of differentiated high-grade thyroid carcinomas: an institutional experience. Endocr Pathol. (2023) 34:287–97. doi: 10.1007/s12022-023-09778-w 37515661

[17] Cracolici V . No longer well-differentiated: diagnostic criteria and clinical importance of poorly differentiated/high-grade thyroid carcinoma. Surg Pathol Clinics. (2023) 16:45–56. doi: 10.1016/j.path.2022.11.003 36739166

[18] Seethala RR Baloch ZW Barletta JA Khanafshar E Mete O Sadow PM . Noninvasive follicular thyroid neoplasm with papillary-like nuclear features: a review for pathologists. Mod Pathol. (2018) 31:39–55. doi: 10.1038/modpathol.2017.130 29052599

[19] Cracolici V Cipriani NA . High-grade non-anaplastic thyroid carcinomas of follicular cell origin: a review of poorly differentiated and high-grade differentiated carcinomas. Endocr Pathol. (2023) 34:34–47. doi: 10.1007/s12022-023-09752-6 36692728

[20] Nikiforov YE Baloch ZW Hodak SP Giordano TJ Lloyd RV Seethala RR . Change in diagnostic criteria for noninvasive follicular thyroid neoplasm with papillarylike nuclear features. JAMA Oncol. (2018) 4:1125–6. doi: 10.1001/jamaoncol.2018.1446 29902314 PMC6584712

[21] Juhlin CC Mete O Baloch ZW . The 2022 WHO classification of thyroid tumors: novel concepts in nomenclature and grading. Endocrine-Related Cancer. (2023) 30:e220293. doi: 10.1530/erc-22-0293 36445235

[22] Masui T Yane K Ota I Kakudo K Wakasa T Koike S . Low Ki-67 labeling index is a clinically useful predictive factor for recurrence-free survival in patients with papillary thyroid carcinoma. J Pathol Trans Med. (2025) 59:115–24. doi: 10.4132/jptm.2024.11.08 39962924 PMC12010870

[23] Erdian DN Ham MF Khoirunnisa D Harahap AS . High Ki-67 labeling index correlates with aggressive clinicopathological features in papillary thyroid carcinoma: a retrospective study. Thyroid Res. (2025) 18:54. doi: 10.1186/s13044-025-00265-4 41174793 PMC12579418

[24] Boudina M Zisimopoulou E Xirou P Chrisoulidou A . Aggressive types of Malignant thyroid neoplasms. J Clin Med. (2024) 13:6119. doi: 10.3390/jcm13206119 39458070 PMC11508432

[25] Raymond P Klein M Borson-Chazot F . Summary and update on the management of differentiated thyroid cancer in 2023. Annales d'endocrinologie. (2024) 85:110–117. doi: 10.1016/j.ando.2023.11.007 38316254

[26] Schipor S Manda D Ceausu M . Aggressive thyroid carcinomas clinical and molecular features: a systematic review. Int J Mol Sci. (2025) 26:5535. doi: 10.3390/ijms26125535 40564998 PMC12192656

