# Supplementary figures and images for: Clinicopathological, proliferative, molecular, and prognostic characteristics of differentiated high-grade thyroid carcinoma: a multicenter retrospective study
Source: Front Oncol. 2026 Jul 8;16:1874863. doi: 10.3389/fonc.2026.1874863 (PMC13388139; doi:10.3389/fonc.2026.1874863)

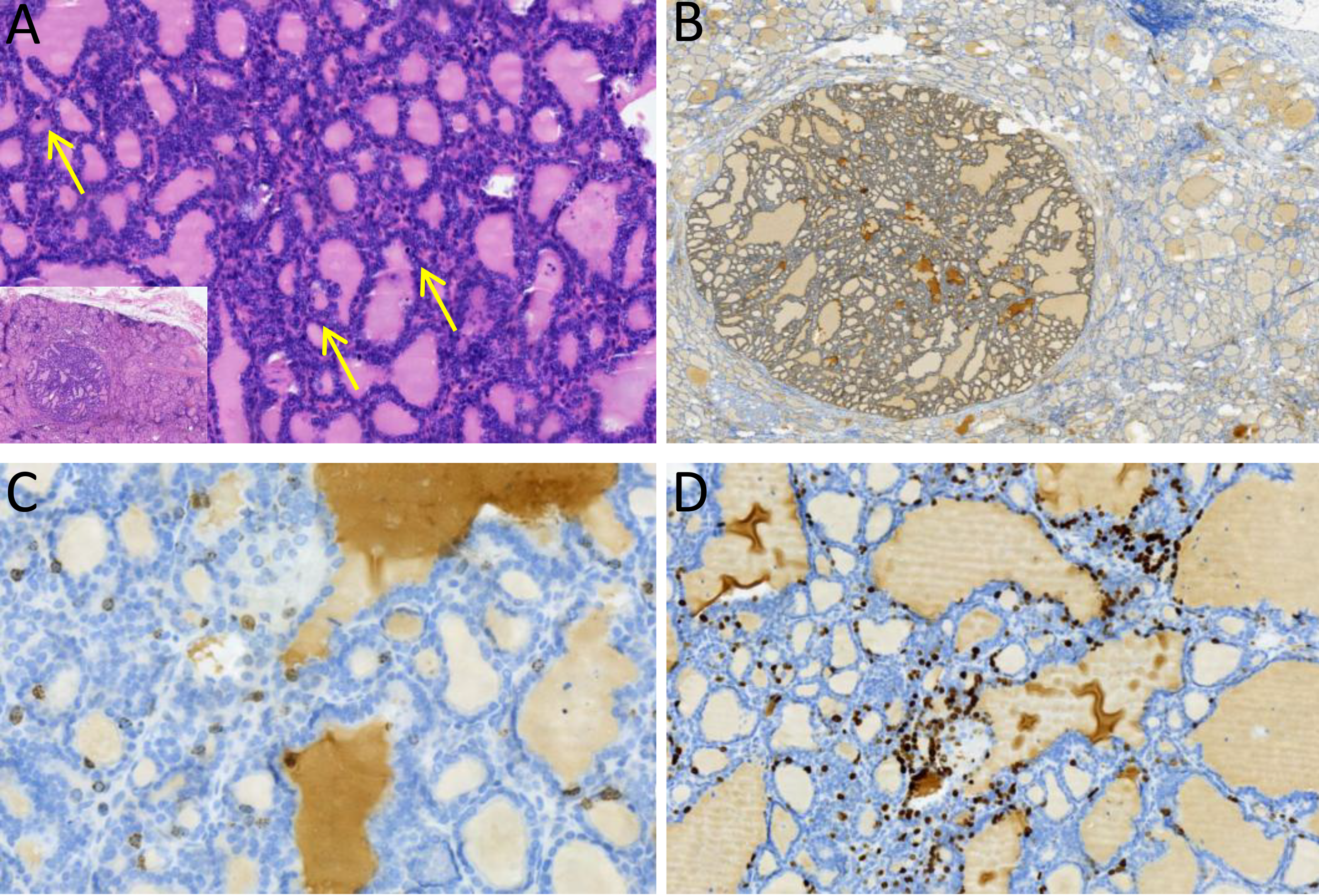

Supplement: Supplementary Figure 1 — Histopathological features of the 24-year-old patient with differentiated high-grade thyroid carcinoma (DHGTC). (A) H&E staining showing frequent mitotic figures at ×200 magnification, with a low-power inset view (×20) demonstrating that the tumor was confined within the thyroid lobe. (B) Diffuse positive CK19 expression by immunohistochemistry. (C) PHH3 staining demonstrating numerous mitotic figures in a high-power field (×400). (D) Ki-67 staining showing a relatively high proliferative index. [file Image1.tif]
